# Is the tryptophan codon of gene *vif* the Achilles’ heel of HIV-1?

**DOI:** 10.1371/journal.pone.0225563

**Published:** 2020-06-22

**Authors:** Fabiola Villanova, Marta Barreiros, Élcio Leal

**Affiliations:** Institute of Biological Sciences, Federal University of Pará, Belém, PA, Brazil; University Hospital Tuebingen, GERMANY

## Abstract

To evaluate the impact of hypermutation on the HIV-1 dissemination at the population level we studied 7072 sequences HIV-1 gene *vif* retrieved from the public databank. From this dataset 854 sequences were selected because they had associated values of CD4+ T lymphocytes counts and viral loads and they were used to assess the correlation between clinical parameters and hypermutation. We found that the frequency of stop codons at sites 5, 11 and 79 ranged from 2.8x10^-4^ to 4.2x10^-4^. On the other hand, at codons 21, 38, 70, 89 and 174 the frequency of stop codons ranged from 1.4x10^-3^ to 2.5x10^-3^. We also found a correlation between clinical parameters and hypermutation where patients harboring proviruses with one or more stop codons at the tryptophan sites of the gene *vif* had higher CD4+ T lymphocytes counts and lower viral loads compared to the population. Our findings indicate that A3 activity potentially restrains HIV-1 replication because individuals with hypermutated proviruses tend to have lower numbers of RNA copies. However, owing to the low frequency of hypermutated sequences observed in the databank (44 out of 7072), it is unlikely that A3 has a significant impact to curb HIV-1 dissemination at the population level.

## 1. Introduction

The apolipoprotein mRNA editing enzyme catalytic polypeptide-like 3 (APOBEC3; A3) proteins are a family of seven cytidine deaminases (A3A, A3B, A3C, A3D, A3F, A3G, and A3H) that restrict certain lentiviruses, retrotransposons, hepatitis B virus and human papillomavirus [1–4]. Many studies focused on the genes A3F, A3G, and A3H because of their innate defense ability to restrict the replication of the HIV-1 [5–21]. Particularly, A3F and A3G are incorporated into viral particles and during reverse transcription, within newly infected cells; by deamination, these proteins alter C in the viral minus-strand DNA to U. This activity of A3 is termed hypermutation because it induces high rates of G-to-A mutation in the newly synthesized plus-strand of viral DNA [2]. Besides, A3F and A3G inhibit the HIV-1 life cycle curbing the reverse transcription and the integration [2, 5, 22]. The hypermutation activity of A3 proteins is highly dependable of the nucleotide context. Particularly, A3G mutates primarily TGGs when this codon is followed by a G (TGGG). On the other hand, A3F, A3D, and A3H mutate TGG into the TGA stop codon when the TGG codon is followed by an A (TGGA) [1, 2, 8].

HIV-1 counteracts the antiviral property of A3 proteins by the activity of the viral [16, 21, 23–25] infectivity factor (Vif). The Vif proteins assemble with viral-specific E3 ubiquitin ligase by interacting with the cellular Cullin5 (Cul5)-ElonginB-ElonginC proteins, inducing ubiquitination of A3G and consequent degradation by the proteasomal complex [17,21,23–25].

It has been proposed that hypermutation is not enough to repress HIV-1 infection within host because proviruses with varying amounts of G→A mutations are commonly observed in the cells of infected individuals [10, 26]. So, it is reasonable that when G→A mutations are ineffective in neutralizing viral genomes, A3G activity can actually increase HIV-1 diversification [9, 27].

In this work, we used 7042 sequences to estimate the rate of stop codons at tryptophan (TGG) residues of the *vif* gene. By assuming that A3 hypermutations are context-dependent and RT mutations are context-independent this approach enables us to distinguish the impact of A3 hypermutation from the RT activity in the *vif* gene.

We found that patients harboring proviruses with one or more stop codons at the tryptophan sites of the gene *vif* had higher CD4+ T lymphocytes counts and lower viral loads compared to the population.

## 2. Materials and methods

### 2.1. Sequence processing

To evaluate the impact of hypermutation to the HIV-1 dissemination at the population level we studied 7072 sequences of the *vif* gene obtained from the public HIV databank at the Los Alamos National Laboratory (https://www.hiv.lanl.gov). From this dataset 854 sequences that had associated values of CD4+ T lymphocytes counts were selected to assess the correlation between clinical parameters and hypermutation. Sequence alignment and editing. The sequences were aligned using the ClustalX program [28]. In addition, the SE-AL program, version 2.0 (Department of Zoology, Oxford University; http://tree.bio.ed.ac.uk/software/seal/) was used to edit the alignment in order to keep all reading frames opened.

### 2.2. Mutation rates and statistical analysis

To compute the stop codons induced by A3 proteins we used the same approach described by Cuevas et al., 2015. Briefly, it is well characterized by the preferential targets of distinct A3 proteins while editing the minus strand of viral DNA [27]. The A3G protein converts GG to AG primarily in the GGG triplet, thus in this context tryptophan codons (TGG) can be converted to the stop codons TAG or TGA. On the other hand, A3F/D/H convert GG to GA in the target GGA, consequently, tryptophan codons (TGG) will be converted into TGA stop codons. Following this conception, it is possible to estimate the amount of A3 mutations compared to the baseline mutations induced by the reverse transcriptase RT. Since *vif* gene of HIV-1 is essential to the life cycle of the virus and the protein has eight canonical tryptophan codons at the sites 5, 11, 21, 38, 70, 79, 89 and 174 we counted the number of stop codons in each of these sites [7, 19, 24, 25]. The Bayesian independent Welch test was used to correlate clinical data and mutations rates. All statistical tests were performed using JASP software v.0.11.1 (https://jasp-stats.org/). Boxplots were constructed using R software v 3.5.1 (www.r-project.org).

## 3. Results

### 3.1. Rates of stop codons

**Fig 1:**
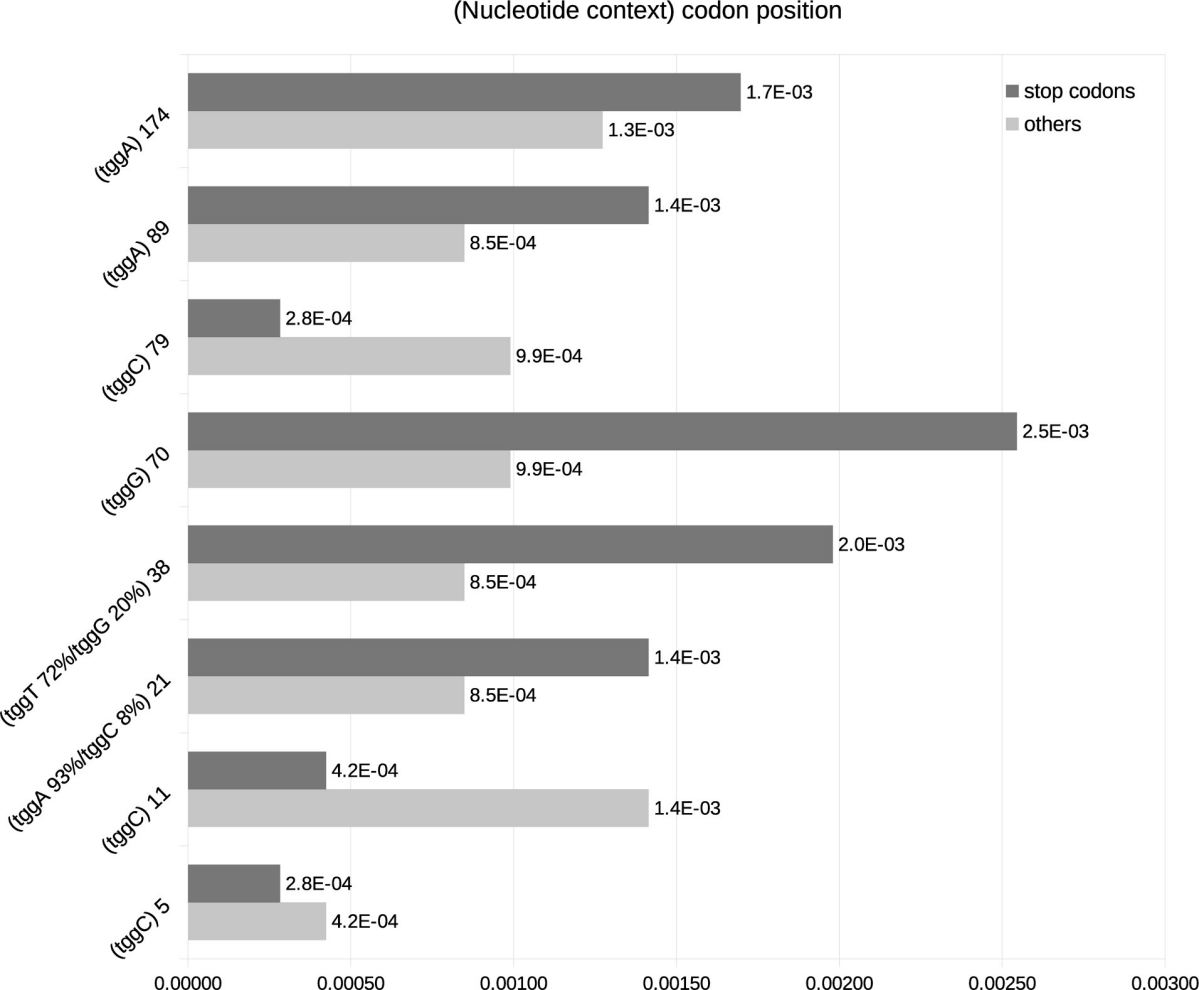
Mutation rates in tryptophan codons of the *vif* gene of HIV-1. At each tryptophan site (TGG) of the gene *vif* the number of amino acid changes was estimated. Dark gray horizontal bars indicate the number of changes from TGG to stop codons (*i*.*e*., TAA, TAG, TGA) and light gray bars represents changes from TGG to any other codons such as AGG, TTG, TCA, etc. The next nucleotide after the TGG is shown in each codon. These nucleotides determine the context target by the human proteins A3 protein (see next figure). Codons 21 and 38 have distinct percentages of a certain nucleotide that is indicated in the figure.

Estimates of mutations rates indicated there are variable (i.e., 83 and 70) and more conserved (i.e., 5) sites in vif (Fig 1). The frequency of stop codons at sites 5, 11 and 79 ranged from 2.8x10^-4^ to 4.2x10^-4^ while at codons 21, 38, 70, 89 and 174 the frequency of stop codons ranged from 1.4x10^-3^ to 2.5x10^-3^. The differences in mutation rates induced by the A3 (dark gray bars in Fig 1) and by the RT (light gray bars in Fig 1) revealed that sites not targeted by A3 proteins (i.e., 5, 11 and 79) were those with lower mutations rates. Conversely, the sites 38 and 70, which are targets of the A3G (at this site TGG is followed by G; TGGG), had higher rates (Fig 1). It is worth mentioning that in site 70 of the Vif all patients presented the context TGGG meanwhile in site 38 the context TGGG was presented in 205 of patients while TGGT was present in 70%. The context TGGT can be mutated either by the A3 activity, converting TGGT into TAGT, or by the RT activity, converting TGGT into TGAT. Besides, at the sites 89 and 174 the tryptophan codon TGG is followed by A, this context (TGGA) is targeted by the A3D/F/H proteins. The above results showed that tryptophan sites 5, 11 and 79, not targeted by A3 proteins, are those with lower rates of stop codons. Conversely, sites targeted by A3 proteins have higher rates of stop codons.

### 3.2. Rates of other mutations

**Fig 2:**
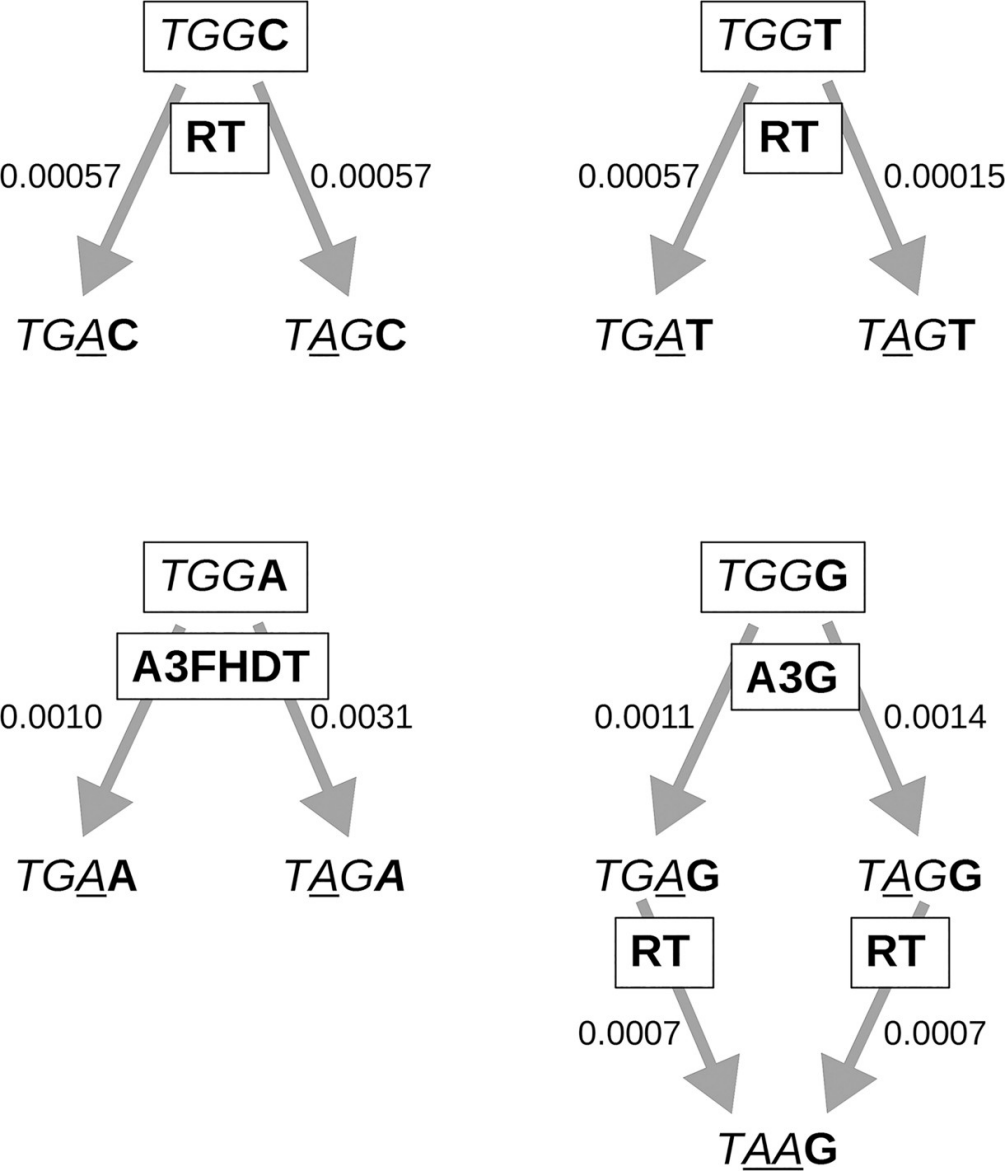
The context associated mutation rates of apobec3. The Context refers to cDNA sequences edited by members of apobec3 (A3) protein family. The human A3G protein targets the CC sequences on minus-strand cDNA, thus causing GG to AG mutations in the positive strand of HIV. A3G mutates TGG codons mainly to TAG stop codon when the TGG codon is followed by a G (TGGG). A3F/H/D mutate TGG to stop codons when TGG is followed by an A (TGGA). Reverse transcriptase has no preferences and can change TGG into any codon, including stop codons. Arrows indicate a mutation from TGG to a certain stop codon and numbers are the estimated mutation rates of stop codons. Boxes close to the arrows indicate the enzyme likely associate with a mutation indicated by the arrow. RT = reverse transcriptase. TGG = Tryptophan, TGA, TAA, TGA = Stop codons.

We also estimated the overall mutation rates considering the TGG context regardless of the position it was located in the vif protein. These measurements were summarized in Fig 2 that shows that mutations from TGG (tryptophan codon) to the stop codons TGA or TAG are higher when the tryptophan codon is followed by Gs or As (TGGG). Tryptophan codons that are targeted by A3 proteins (i.e., TGGG or TGGA) have rates of 1x10^-3^ to 3.1x10^-3^ while codons target by the RT has rates of 2x10^-4^ to 6x10^-4^.

### 3.3. Stop codons and clinical status

**Fig 3:**
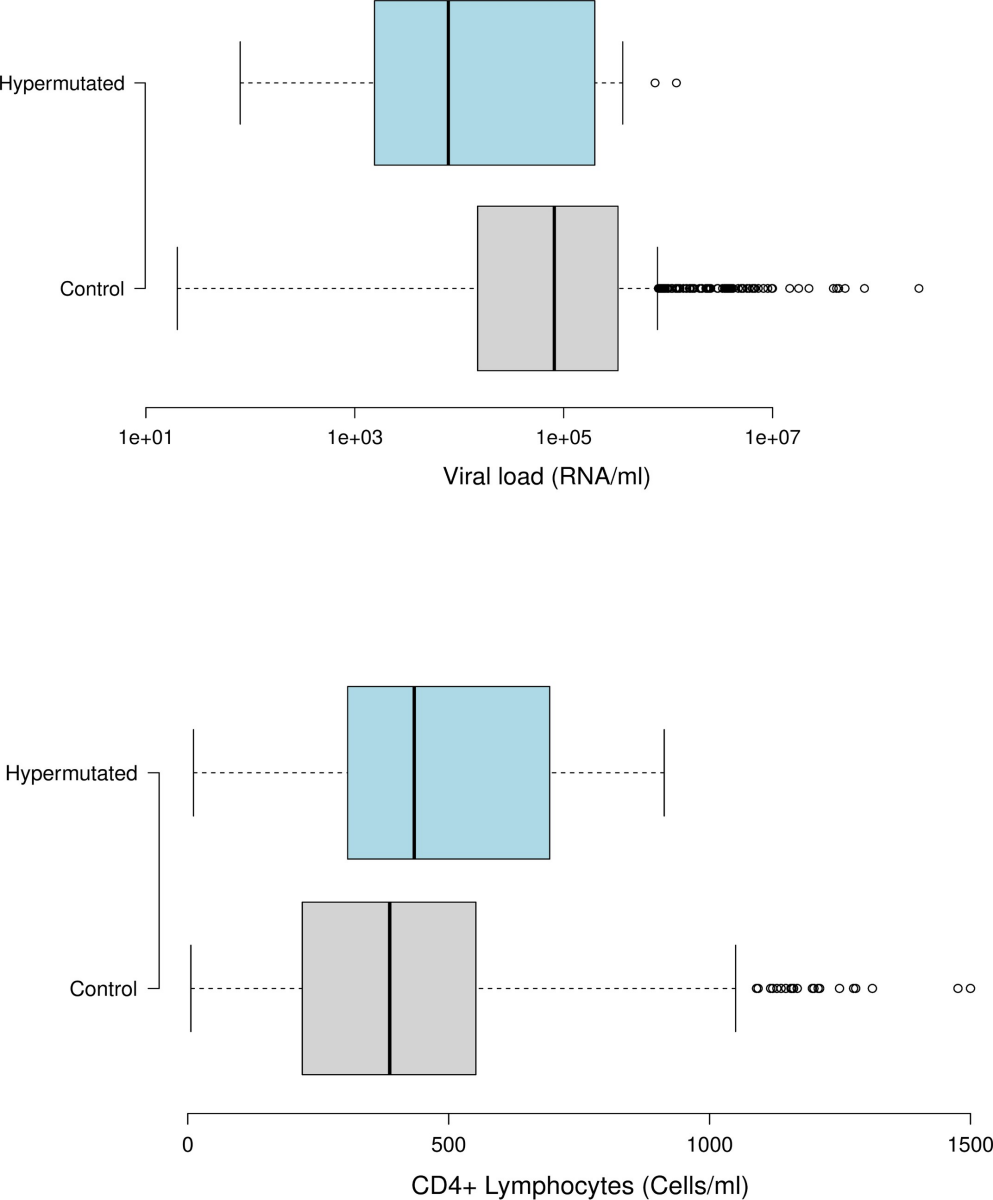
RNA levels and CD4+ cell counts. Comparisons of RNA levels per ml (upper boxes) and counts of CD4+ cells per ml (lower boxes) between the sequences with stop codons at the tryptophan (grey boxes) and sequences without stop codons (white boxes). In the boxes the center lines show the medians; box limits indicate the 25th and 75th percentiles. Whiskers extend 1.5 times the interquartile range from the 25th and 75th percentiles, outliers are represented by open dots. RNA levels (upper boxes) are shown in log scale.

To assess the correlation between clinical parameters and mutations we used 854 sequences that had associated values of CD4+ T lymphocytes counts and viral loads. We found eleven sequences having one or more stop codons at the tryptophan sites of the gene *vif*. Notably, these sequences presented lower viral loads (posterior probability = 0.097) and higher levels of CD4+ lymphocytes (posterior probability = 0.071) compared with the overall values of 854 dataset (Table 1). The median viral load in patients with hypermutation was equal to 7,864.00 copies/mL (variance = 1.624E11) with a mean of 216,392.36 copies/mL, but in patients without hypermutation the median was 50,709.00 copies/mL (variance = 2.865E11) and mean of 225,917.78 copies/mL. The median CD4 + T lymphocytes for the group with hypermutation was 434.00 (variance = 67968.82) cells/mm^3^ and mean of 485.72 cells/mm^3^, while the samples without hypermutation median CD4 + T cells was 403.00 (variance = 65505.90) cells/mm^3^ and mean of 434.61 cells/mm^3^ (Fig 3).

**Table 1:** Hypermutation in *vif* gene and clinical parameters of HIV-1 infected patients.

***Viral loads in hypermutated and non hypermutated vif gene of HIV-1***		
**Hypothesis**	**Bayes factor**	**Posterior probability**
H0:equal		0.794
H1:Bigger	7.239	0.110
H2:Smaller	8.192	0.097
***CD4 levels in hypermutated and non-hypermutated vif gene of HIV-1***		
H0: Equal		0.726
H1: Bigger	10.232	0.071
H2: Smaller	3.577	0.203

Bayesian informative hypotheses evaluation (Independent Samples Welch's T-Test). The null hypothesis H0 (equal hypermutated versus non-hypermutated means) is tested against H1 (first mean larger than second mean) and H2 (first mean smaller than second mean). The posterior probabilities are based on equal prior probabilities.

## 4. Discussion

The amount of A3G hypermutation varies considerably along HIV-1 genome and this gradient of G-to-A substitutions correlates with the time the minus strand remains as a single-stranded molecule during replication [26]. One consequence of the hypermutation gradient is that some genes are more affected than others. The *vif* gene has the lowest amount of A3G-associated mutation compared to other HIV-1 genes [27]. Vif also has tryptophan residues (W) at the specific positions 5, 11, 21, 38, 70, 89 and 174 that are involved in A3G and A3F binding. These codons will be target by the A3 activity and the TGG codon will be changed into a stop codon (e.g., TAG, TGA, TAA). Equally, the TGG codon will be targeted by RT activity converting it into stop codons and also into others codons such as TTG, TGT, AGG, etc.

Vif has some conserved residues, notably in the motifs ^14^DRMR^17^, ^21^WK/NSLVK^26^, ^40^YRHHY^44^ and ^161^PPLP^164^. For example, we found the overall 16% of conserved residues in 317 HIV-1 subtype B vif sequences in Brazil [19]. This lower diversity in some residues has been related to the very strong purifying selection detected on this viral protein, thus indicating that vif is essential to the HIV life cycle [4,7,19,21,23]. While hypermutation induced by A3G activity is a natural barrier against retroviruses it is not enough to restrain HIV-1 infection. Since HIV-1 infection is characterized by multiple strains forming a quasispecies, then it is likely that hypermutated strains can benefit from circulation of or even reservoir viruses in distinct tissues. It is likely that A3G activity can actually increase HIV-1 diversification when G-to-A hypermutation is ineffective in neutralizing all viral genomes within a host [9].

Colson et al., [29] showed that in long-term non-progressors patients A3 activity is able to restrain HIV-1 replication by changing of tryptophan (TGG) codons into stop codons (TAG/TGA) mainly on the gene *vif*. However, the effect of hypermutation to the spread of HIV-1 is not known yet. We studied this subject by using the tryptophan codons of Vif as a proxy to evaluate the A3 activity to potentially reduce the chances of HIV spread between individuals. Our analysis indicated a correlation between clinical parameters and hypermutation where patients harboring proviruses with one or more stop codons at the tryptophan sites of the gene *vif* had higher CD4+ T lymphocytes counts and lower viral loads compared to the population. We found a correlation between clinical parameters and hypermutation in patients harboring proviruses with one or more stop codons at the tryptophan sites of the gene *vif* had higher CD4+ T lymphocytes counts and lower viral loads compared to the population. Thus our findings indicate that A3 activity potentially restrains HIV-1 replication because individuals with hypermutated proviruses tend to have a lower number of RNA copies. However owing to the low frequency of hypermutated sequences observed in the databank (44 out of 7072), it is unlikely that A3 has a significant impact to curb HIV-1 dissemination at the population level.

Our findings are in consent with the observation that A3G hypermutation is more frequent among elite controllers [30]. It is also worth to mention that CD4+ T lymphocytes counts are related to selective diversity and hypermutation [11, 13, 31].

## Supporting information

S1 Data (RAR)
